# Methods to Investigate the Global Atmospheric Microbiome

**DOI:** 10.3389/fmicb.2019.00243

**Published:** 2019-02-21

**Authors:** Aurelien Dommergue, Pierre Amato, Romie Tignat-Perrier, Olivier Magand, Alban Thollot, Muriel Joly, Laetitia Bouvier, Karine Sellegri, Timothy Vogel, Jeroen E. Sonke, Jean-Luc Jaffrezo, Marcos Andrade, Isabel Moreno, Casper Labuschagne, Lynwill Martin, Qianggong Zhang, Catherine Larose

**Affiliations:** ^1^Institut des Géosciences de l’Environnement, Univ. Grenoble Alpes, CNRS, IRD, Grenoble INP, Grenoble, France; ^2^Institut de Chimie de Clermont-Ferrand, UMR6096 CNRS–Université Clermont Auvergne-Sigma, Clermont-Ferrand, France; ^3^CNRS UMR 5005, Environmental Microbial Genomics, Laboratoire Ampère, École Centrale de Lyon, Université de Lyon, Ecully, France; ^4^Laboratory for Meteorological Physics (LaMP), Université Clermont Auvergne, Clermont-Ferrand, France; ^5^Géosciences Environnement Toulouse, Centre National de la Recherche Scientifique, Institut de Recherche pour le Développement, Université de Toulouse, Toulouse, France; ^6^Laboratory for Atmospheric Physics, Institute for Physics Research, Universidad Mayor de San Andrés, La Paz, Bolivia; ^7^Department of Atmospheric and Oceanic Sciences, University of Maryland, College Park, MD, United States; ^8^South African Weather Service, Stellenbosch, South Africa; ^9^Key Laboratory of Tibetan Environment Changes and Land Surface Processes, Institute of Tibetan Plateau Research, Chinese Academy of Sciences, Beijing, China

**Keywords:** atmosphere, microorganisms biodiversity, aerobiology, biogeography, protocols, methods, aerosols

## Abstract

The interplay between microbes and atmospheric physical and chemical conditions is an open field of research that can only be fully addressed using multidisciplinary approaches. The lack of coordinated efforts to gather data at representative temporal and spatial scales limits aerobiology to help understand large scale patterns of global microbial biodiversity and its causal relationships with the environmental context. This paper presents the sampling strategy and analytical protocols developed in order to integrate different fields of research such as microbiology, –omics biology, atmospheric chemistry, physics and meteorology to characterize atmospheric microbial life. These include control of chemical and microbial contaminations from sampling to analysis and identification of experimental procedures for characterizing airborne microbial biodiversity and its functioning from the atmospheric samples collected at remote sites from low cell density environments. We used high-volume sampling strategy to address both chemical and microbial composition of the atmosphere, because it can help overcome low aerosol and microbial cell concentrations. To account for contaminations, exposed and unexposed control filters were processed along with the samples. We present a method that allows for the extraction of chemical and biological data from the same quartz filters. We tested different sampling times, extraction kits and methods to optimize DNA yield from filters. Based on our results, we recommend supplementary sterilization steps to reduce filter contamination induced by handling and transport. These include manipulation under laminar flow hoods and UV sterilization. In terms of DNA extraction, we recommend a vortex step and a heating step to reduce binding to the quartz fibers of the filters. These steps have led to a 10-fold increase in DNA yield, allowing for downstream omics analysis of air samples. Based on our results, our method can be integrated into pre-existing long-term monitoring field protocols for the atmosphere both in terms of atmospheric chemistry and biology. We recommend using standardized air volumes and to develop standard operating protocols for field users to better control the operational quality.

## Introduction

Biological particles are known to represent a significant fraction (∼20–70%) of the total number of aerosols > 0.2 μm, with large spatial and temporal variations (Matthias-Maser and Jaenicke, 1995; Graham et al., 2003; Jaenicke, 2005; Huffman et al., 2012). Among these, microorganisms are of particular interest in fields as diverse as epidemiology, including phytopathology (Morris et al., 2007), bioterrorism, forensic science and public health (Galán Soldevilla et al., 2007), and environmental sciences, like microbial ecology (Monteil et al., 2014; Mayol et al., 2017; Michaud et al., 2018), meteorology and climatology (Sesartic et al., 2012; Pouzet et al., 2017). More precisely concerning the latter, airborne microorganisms contribute to the pool of particles nucleating the condensation and crystallization of water and they are thus potentially involved in cloud formation and in the triggering of precipitation (Morris et al., 2014; Fröhlich-Nowoisky et al., 2016). Additionally, viable microbial cells act as chemical catalyzers interfering with atmospheric chemistry (e.g., Vaïtilingom et al., 2013). The constant flux of bacteria from the atmosphere to the Earth’s surface due to precipitation and dry deposition can also affect global biodiversity, but they are rarely taken into account when conducting ecological surveys (Hughes and Convey, 2010; Bar-On et al., 2018; Leyronas et al., 2018; Reche et al., 2018). As stressed by these studies attempting to decipher and understand the spread of microbes over the planet (e.g., Burrows et al., 2009a; Bowers et al., 2013; Morris and Sands, 2017; Šantl-Temkiv et al., 2018), concerted data are needed for documenting the abundance and distribution of airborne microorganisms, including at remote and altitudes sites.

Airborne bacteria are emitted by most Earth surfaces (plants, oceans, land, and urban areas) to the atmosphere via a variety of mechanical processes such as aeolian soil erosion, sea spray production, or mechanical disturbances including anthropogenic activities (e.g., Joung et al., 2017; Michaud et al., 2018). Due to their relatively small size (the median aerodynamic diameter of bacteria-containing particles is around 2–4 μm (Despres et al., 2012), these can then be transported upward by turbulent fluxes (Carotenuto et al., 2017) and carried by wind to long distances. As a consequence, bacteria are present in the air up to at least the lower stratosphere (Wainwright et al., 2006; DeLeon-Rodriguez et al., 2013; Smith et al., 2018). Given that the atmosphere is a large conveyor belt that moves air over thousands of kilometers, microorganisms are disseminated globally (Smith et al., 2012, 2013; Griffin et al., 2017). Airborne transport of microbes is therefore likely pervasive at the global scale, yet there have been only a limited number of studies that have looked at the spatial distribution of microbes across different geographical regions (e.g., Barberán et al., 2015; Griffin et al., 2017). One of the main difficulties is linked with the low microbial biomass associated with a high diversity existing in the atmosphere outdoor (∼10^2^–10^5^ cells/m^3^; e.g., Burrows et al., 2009b; Bowers et al., 2013; Amato et al., 2017), thus requiring reliable sampling procedures and controls. Furthermore, the site location and its environmental specificities have to be accounted for to some extent by considering chemical and meteorological variables (Deguillaume et al., 2014).

While these studies have led to novel findings regarding the link that may exist between airborne bacteria and their source and receptacle environments, the lack of uniform sampling and analysis methodology weaken the conclusions that can be drawn from independent studies. Here we aimed to provide sample collection and preparation methods intended to generate reproducible data, applicable to most sampling locations. This should allow the investigation of long-range transport, surface ecosystem interconnectivity and distribution of microorganisms in relation to meteorological and chemical contexts. Here, we present a method that allows simultaneous -sampling for chemical and microbiological characterization of aerosols, which can be deployed for long term monitoring at atmospheric observation sites throughout the planet. This has only been carried out previously in urban areas and the methods were not developed or optimized for non-urban environments (Bertolini et al., 2013) or for subsequent chemical analysis (Jiang et al., 2015; Luhung et al., 2015). The main objectives were to: (1) define appropriate sampling methods and duration (2) set up quality controls in order to improve the detection limit for various chemical species (3) improve DNA extraction methods from low biomass samples (4) ensure data intercomparability and (5) develop simplified experimental workflows that can easily be carried out by non-specialist onsite technical staff. These protocols were tested at 10 distinct sites covering various geographic regions of the globe. The need for a coordinated network for global monitoring of aerobiology has recently been identified in a number of recently published studies (Smith, 2013; Pearce et al., 2016; Cáliz et al., 2018), however, a standardized sampling method has yet to be proposed. Based on our results, our method can be integrated into pre-existing long-term monitoring field protocols for the atmosphere both in terms of atmospheric chemistry and biology, and could be included in future projects.

## Methods

### Experimental Strategy

A variety of methodologies for bioaerosol sampling, including passive sampling, filtration and impaction techniques exist (Haddrell and Thomas, 2017), however these have yet to be harmonized for concerted studies. In order to deal with the equipment available at most international monitoring stations, a sampling protocol that could be carried out by non-specialized personnel using on-site sampling equipment needed to be designed; these conditions constrained the choice of our bioaerosol sampling strategy toward high-volume samplers (high air flow-rate) on large diameter/size quartz fiber filters. Sampling time as well as DNA extraction protocols were improved for ensuring obtaining sufficient biological material for analyses from these types of filters, while maintaining the sampling time as short as possible for allowing detecting variations in connection with environmental variables. The sampling strategy and protocol development are outlined in the following section.

### Filter Selection and Development of Sterilization Protocols

The analysis for chemical compounds and elements [e.g., elemental carbon (EC) and organic carbon (OC)] requires the combustion of a quartz fiber filter, which constrained the choice of sampling material. Depending on the sampler model, two filters sizes were used (5.9′′ round filter and 8′′ × 10′′ rectangular types) with filtration surface areas of 163 and 526 cm^2^, respectively. Several sterilization methods were tested to improve the biological quality of the filter without altering the detection limits of the chemical parameters. A standard method for atmospheric chemistry protocols is to dry heat the quartz filters at 500°C for at least a few hours (Jaffrezo et al., 2005). We tested additional heating and sterilization steps in laminar flow hoods or UV-exposure (10 min) on both filters and storage material. By adding these supplementary steps, we were able to significantly reduce the background OC concentrations of our blank filters as compared to the standard method (standard method OC concentration = 0.93 ± 0.35 μg/cm^2^ of filter, new method OC concentration = 0.55 ± 0.26 μg/cm^2^, *p* = 0.0009, Student *T*-test).

Based on our results, the following protocol was defined: filters were heated to 500°C for 8 h in order to remove traces of organic carbon including DNA. Filters were then handled within a laminar flow hood (UV sterilized, 254 nm, PSM – ESI FLUFRANCE BIOCYT 120, 10 min on each filter side) and individually stored in a folded aluminum foil and a thermally-sealed plastic (PE) bag or a zip-lock bag. All the material including foils, plastic bags, tweezers that would be in contact with the filters was UV-sterilized (2 J/cm2 for 2 min, 254 nm, CrossLinker, Bio-Link BLX). The filter holders were also UV-sterilized and stored individually in sterile bags. At the sampling sites, the field operators were trained and were given a detailed protocol (see Supplementary Information for detailed protocol) in order to replace and handle the filters properly at defined sampling times. After collection, filters were sealed in a folded sterile aluminum foil and plastic bags and stored at -20°C. At most sites, no microbiological safety cabinet was available; thus clean benches were made using pre-UV-sterilized plastic sheets in order to minimize contamination. All the sterile material was provided in sufficient quantity to the field operators. After sampling, filters (exposed and controls) were shipped to France for analysis from each sampling site at below zero temperature.

### Optimization of DNA Extraction

While quartz filters have been used for microbial studies in the air (Després et al., 2007; Smith et al., 2012; Jiang et al., 2015), limitations exist regarding the integrity of the samples collected [DNA degradation, cell mortality (Luhung et al., 2015)]. In addition, classic DNA extraction methods need to be improved for investigations in remote sites with low biomass. Since we were constrained by the choice of quartz filters for chemical analysis, we tested different extraction protocols in order to optimize DNA extraction yield from these filters. We tested different DNA extraction kits developed for environmental samples (e.g., DNeasy PowerWater, DNeasy PowerSoil and DNeasy Blood and Tissue kits from Qiagen). To do so, we set up a size selective high volume air sampling instrument (DIGITEL) equipped with a PM10 size-selective inlet in order to collect airborne particulate matter smaller than 10 μm (cut-off aerodynamic diameter) from the roof of the laboratory in Grenoble (France) to mimic field conditions. An atmospheric sample was collected for 24 h on a large filter. Nine sub-samples were collected from this filter and DNA was extracted according to the manufacturer’s instructions with the following modification: after the lysis step, the lysate was centrifuged in a syringe for 4 min at 1000 rcf to drain filter debris that tended to absorb the lysis solution. This additional centrifugation step increased lysate volume recovery by more than five-fold, potentially increasing DNA recovery. DNA concentrations were compared following quantification with a fluorometric method (Qubit dsDNA HS Assay Kit from Thermo Fisher Scientific, manufacturer’s instructions on 10 μL of sample) and 16S rRNA gene copy numbers were compared following quantification using qPCR. Briefly, the V3 region of the 16S rRNA gene was amplified using the SensiFast SYBR No-Rox kit (Bioline) and the following primers sequences: Eub 338f 5′-ACTCCTACGGGAGGCAGCAG-3′ as the forward primer and Eub 518r 5′-ATTACCGCGGCTGCTGG-3′ as the reverse primer (Fierer et al., 2005) on a Rotorgene 3000 machine (Qiagen). The reaction mixture of 20 μL contained 10 μL of SYBR master mix, 2 μL of DNA and RNAse-free water to complete the final 20 μL volume. The 2-step qPCR program consisted of an initial step at 95°C for 2 min for enzyme activation, then 35 cycles of 5 s at 95°C and 20 s at 60°C for hybridization and elongation, respectively. A final step was added to obtain a denaturation from 55 to 95°C with increments of 1°C. The amplicon length was around 200 bp. PCR products obtained from DNA from a pure culture of *E. coli* were cloned in a plasmid (pCR^TM^2.1-TOPO^®^ vector, Invitrogen) and used as standard after quantification with the Broad-Range Qubit Fluorometric Quantification (Thermo Fisher Scientific).

The DNA concentrations measured were not significantly different among the three DNA extraction kits (Soil 0.008 ± 0.005 ng/μL, Water 0.010 ± 0.005 ng/μL, Tissue 0.012 ± 0.002 ng/μL, *p* > 0.22, One-way ANOVA, Tukey tests). However, the number of 16S rRNA gene copies per cubic meter of air was on average ten times higher with DNeasy PowerWater and PowerSoil kits than it was with DNeasy Blood & Tissue kit. Differences in extraction efficiency have been previously observed for several metagenomic and taxonomic studies and it is suggested that a variety of methods be tested to optimize results before studying new environments (Delmont et al., 2012; Zielińska et al., 2017). Based on these results and for practical reasons, we chose to use the DNeasy PowerWater kit. The DNeasy PowerBead tubes of the DNeasy PowerWater kit are 5 mL tubes (compared to the 2 mL PowerBead tubes of the DNeasy PowerSoil kit) allowing extraction of a larger filter surface.

**Figure 1:**
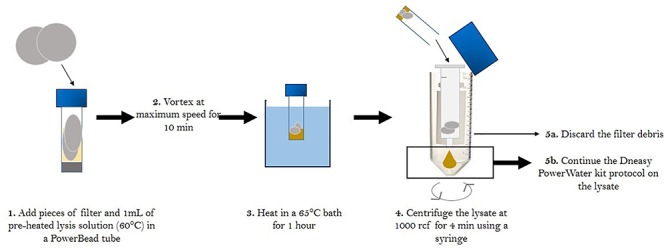
Summary of the modified DNA extraction protocol developed for quartz filters.

The second series of extraction tests were carried out to determine whether the filter handling protocol had an effect on extraction efficiency. We tested four different treatments in triplicate: blank filters (processed and brought to the field but unexposed), filters that were passively exposed to the atmosphere for 5 min, filters that collected atmospheric samples during 24 h without heat treatment and filters that collected atmospheric samples during 24 h with heat treatment. DNA was extracted from filters using the protocol outlined above and quantified using Qubit. Based on the results of our test, heating the quartz filters at 500°C for 8 h before sampling has a significant impact on DNA extraction efficiency and reduced yield by up to 10-fold (24 h no heating 0.33 ± 0.16 ng/μL, *n* = 3, 24 h heating 0.038 ± 0.005 ng/μL, *n* = 3, *p* < 0.05, student *t*-test), but this is a critical step to ensure that trace carbon is removed from the filters prior to sampling. Therefore, we needed to further optimize our method to increase DNA extraction efficiency from heat sterilized quartz filters. Different options were tested, such as modifying the pH by addition of 1 M CaCO3 and shaking, as described in Bertolini et al. (2013), as well as adding a sonication step (Luhung et al., 2015), Neither of these methods increased yield significantly and we preferred to avoid adding a solution to our samples. Considering that high temperatures help desorbing DNA from the silica in the quartz filter (Vandeventer et al., 2013), we also tested the efficiency of a 1-h thermal treatment at 65°c during the lysis step (Jiang et al., 2015;Luhung et al., 2015) on DNA yield using eleven samples. We obtained four to five times higher DNA concentrations and 100–1000 times higher 16s rRNA gene copies per cubic meter of air using this optimized lysis technique. The final protocol is summarized in Figure 1 and was applied to all the filters collected during the sampling campaign.

### Optimization of Sampling Time for DNA Analysis

Once the filter treatment and DNA extraction protocols were validated, we carried out different tests to optimize sample collection duration. We used the same sampling set-up on the roof of the laboratory in Grenoble to collect atmosphere samples. Several different sampling times were considered: 5 min, 1, 24, and 72 h. DNA was extracted from filters using the protocol outlined above and quantified using Qubit. Filters analyzed in triplicate at 5 min and 1 h where found to be below the detection limit of the apparatus (0.01 ng/μL). Filters collected at 24 h showed a significantly lower yield than those collected at 72 h (24 h = 0.02 ± 0.01 ng/μL, 72 h = 0.08 ± 0.03 ng/μL, *n* = 3, *p* < 0.05, Student *t*-test), which suggests that sampling duration impacts DNA yield. Based on the results of this test and considering the remoteness of the sites, we decided to sample continuously for 7 days.

### Protocol Testing and Deployment

**Figure 2:**
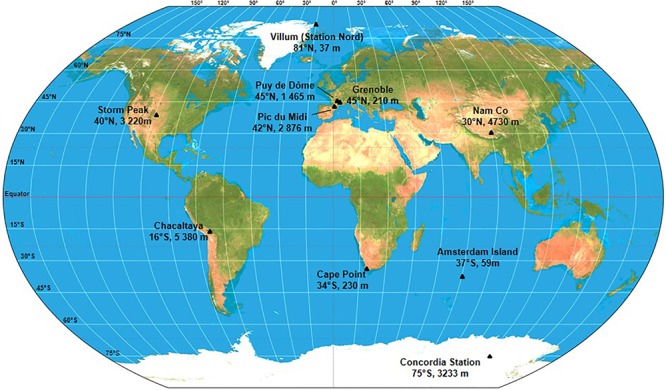
Global distribution of the sampling sites and their respective elevation above sea level.

Ten sites were chosen based on latitudinal positions, known chemical characteristics, historic atmospheric data and logistical support. The characteristics for each site are described in Table 1 and the geographic distribution can be seen in Figure 2. Sites included Arctic and Antarctic stations as well as mid-latitude stations. In order to access information on long range transport of aerosols, dusts and airborne microorganisms, three sites that are frequently in the free troposphere were selected. Temporal variability is an important but poorly understood factor of microbial community diversity (e.g., Bertolini et al., 2013), so we completed the dataset with continuous sampling for more than one entire year at a 1-week resolution at a single reference site: puy de Dôme, France. To avoid snap-shot sampling, each site was sampled for a minimum of 2 months, so manned research sites were selected. At all sampling sites, meteorological parameters, such as wind speed and direction, rainfall, temperature, humidity, air pressure, and solar radiation were systematically recorded. Depending on the site, continuous measurements providing additional information for data interpretation were also collected. These included aerosol properties (size, concentration) and gas concentrations (ambient O_3_, nitrogen species, CO, CO_2_, CH_4_, H_2_O, O_2_, and volatile organic compounds (VOCs). Finally, total gaseous mercury (TGM) concentration was also continuously monitored by our collaborators at Cape Point Station, Nam Co, Amsterdam Island and Villum Research Station.

**Table 1:** Summary of sampling sites characteristics.

								Mean air
								volume per
Samping	Sampling site				Collection period (dd/mm/yyyy)		Sampling duration	filter (SATP approx.)	Number of samples
site code	location	Coordinates and altitude	Site characteristics	Sampler model	Start	Stop			
									
AMS	Amsterdam Island, France	37°47′82′′S 77°33′04′′E 59 m asl	Remote and natural background of marine environments (Sciare et al., 2009)	Custommade PM10 5.9′′ ∅ size	07/09/2016	10/11/2016	1 week	5000 m3	9
CPT	Cape Point Station, South Africa	34°21′26′′S 18°29′51′′E 230 m asl	South Atlantic Ocean, greater Cape Town and other continental sources (Brunke et al., 2010).	Digital DA77 PM10 5.9′′ ∅ size	11/10/2016	05/12/2016	1 week	4600 m3	7
CHC	Chacaltaya, Bolivia	16°20′47′′S 68°07′44′′W 5380 m asl	Tropical free troposphere local urban pollution (Andrade et al., 2015; Rose et al., 2015)	Custommade PM10 5.9” ∅ size	27/06/2016	11/11/2016	1 week ^∗^	2000 m3	16
DMC	Concordia Station, Antarctica	75°06′00′′S 123°19′58′′E 3233 m asl	Cold environment, polar boundary layer (Angot et al., 2016)	TISCH TSP 8′′ × 10′′ size	19/12/2014	31/01/2015	2 weeks	16000 m3	3
GRE	Grenoble, France	45°11′38′′N 05°45′44′′E 210 m asl	Urban air, European air masses	Digital DA77 PM10 5.9′′ ∅ size	30/06/2017	14/09/2017	1 week	4700 m3	10
NCO	Nam Co, China	30°46′44′′N 90°59′31′′E 4730 m asl	Tibetan Plateau, no major anthropogenic sources, cold and dry conditions, intense solar radiation (Yin et al., 2017).	Chinese HV PM10 8′′ × 10′′ size	16/05/2017	28/07/2017	1 week	5300 m3	9
PDD	Puy de Dôme, France	45°46′20′′N 02°57′57′′E 1465 m asl	Urban, oceanic, continental air masses, free troposphere at times, (Sellegri et al., 2003; Vaitilingom et al., 2012; Gabey et al., 2013; Farah et al., 2018)	Custommade PM10 5.9′′ ∅ size	23/06/2016	23/08/2017	1–2 week(s)	10000 m3	53
PDM	Pic du Midi, France	42°56′11′′N 00°08′34′′E 2876 m asl	Mountain top, North Atlantic Ocean, European continent and anthropogenic air masses (Gheusi et al., 2011; Fu et al., 2014, 2016)	TISCH PM10 8′′ × 10′′ size	20/06/2016	04/10/2016	1 week	8000 m3	13
STN	Villum research Station, Station Nord, Greenland	81°34′24′′N 16°38′24′′E 37 m asl	Emission from sea-ice, form the Arctic Ocean and from long-range transport from northern Eurasia (Fenger et al., 2013)	Digital DA77 PM10 5.9′′ ∅ size	20/03/2017	29/06/2017	1 week	5200 m3	13
STP	Storm Peak Laboratory, USA	40°27′18′′N 106°44′38′′E 3220 m asl	Free troposphere, westerly winds, urban pollution during the day (Borys and Wetzel, 1997; Obrist et al., 2008).	TISCH PM10 8′′ × 10′′ size	11/07/2017	04/09/2017	1 week	5700 m3	7

*Column 4 describes the high volume sampler as well as the quartz fiber filter size and type. Column 8 gives the number of filters (samples + blanks) collected at each sampling site. Volume are expressed at standard ambient temperature and pressure (SATP). ^∗^Nighttime only*.

Several brands of size selective high volume air sampling instruments (TISCH, DIGITEL, home-made) were used in the present study. All these samplers are based on the same physical principle. Air was drawn into the sampler and through a large quartz fiber filter by means of a powerful pump, so that particulate material impacted the filter surface. All the samplers, but one (at Concordia station in Antarctica -DMC), were equipped with a PM10 size-selective inlet in order to collect particulate matter smaller than 10 μm (cut-off aerodynamic diameter). The use of the PM10 inlet was an important aspect in order to guarantee that both chemical and microbial data could be compared among the sites and to prevent rain and hydrometeors from reaching the filter and modifying its porosity and air flow rate properties. At DMC, total suspended particles were collected (median aerodynamic diameter of 20 μm approx). Sampling air flow rates were between 30 and 70 m^3^/h (±2%) and collected volumes ranged from 2000 to 10000 m^3^ over a 1-week period (except at DMC, where the samples were collected over a 2-week period). Flow regulation of the pump was controlled with a flow-meter that was regularly checked and calibrated. The total volume of air was subsequently corrected to standard ambient temperature and pressure (SATP, 298K, 101.325 kPa) to standardize air collection at all the sites (Table 1). For some mountain sites (PDM, CHC, STP), we generally sampled at nights in order to limit the sampling of the planetary boundary layer air.

### Quality Control

**Figure 3:**
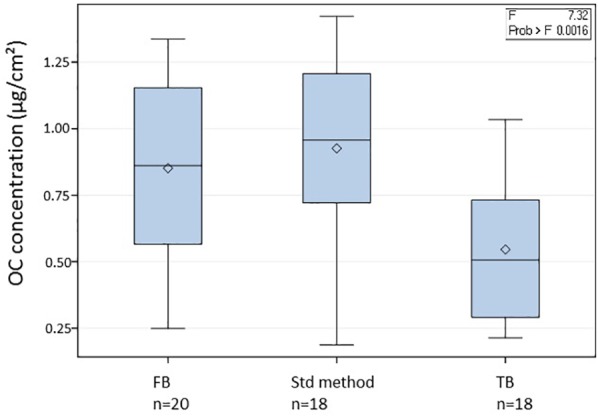
Organic concentrations on different types of filters: field blanks (FB), transport blanks (TB) and filters treated using the standard method (Std method). Significance was tested using One-way ANOVA and Tukey tests.

In addition to the 140 samples, we also collected 38 blank filters named “transportation blanks” (TB, 18 filters) and “field blanks” (FB, 20 filters) in order to monitor and check the quality of the sampling protocol (see Figure 3). The transportation blanks were filters shipped back and forth to the sampling sites but without any manipulation. The field blanks were exposed for 24–72 h without switching on the high-volume sampler and then processed and stored similarly as the actual samples. The field blanks had significantly higher OC concentrations as compared to the transportation blanks, but these were in the same range as the values obtained for blank filters using the standard sterilization technique (without subsequent sterilization steps).

### Chemical Analyses

Elemental carbon (EC), organic carbon (OC), sugar anhydrides and alcohols, major soluble anions and cations as described by Waked et al. (2014) as well as total mercury were systematically analyzed in all the quartz fiber filters (including blank filters). EC and OC were analyzed from a 1.5 cm^2^ punch sample using a thermo optical transmission method on a Sunset Lab analyzer (Birch and Cary, 1996). Punches of 38 mm-diameter from each samples were extracted using ultrapure water under mechanical agitation for a period of 30 min. The extracts were then filtered with 0.22 μm Nucleopore filters before injection in the instruments (Piot et al., 2012). The extracts were used for quantification of sugar anhydrides and alcohols (levoglucosan, mannosan, galactosan, inositol, glycerol, erythriol, xylitol, arabitol, sorbitol, mannitol, trehalose, rhamnose, glucose, fructose, and sucrose) by HPLC-PAD using a set of Methrom columns (MetroSep A Supp 15 and Metrosep Carb1) in a Thermo Scientific^TM^ Dionex^TM^ ICS-5000+ Capillary HPIC^TM^ system. Soluble anions (MSA, SO_4_^2-^, NO_3_^-^, Cl^-^, Ox) and cations (Na^+^, NH_4_^+^, K^+^, Mg^2+^, Ca^2+^) were analyzed by ion chromatography (IC, Dionex ICS3000) on the same extracts. AS/AG 11HC and CS/CG 12A columns were used for anions and cations analyses, respectively. Finally, twenty-five low molecular weight organic acids (C3-C9) were analyzed from the same extracts by LC-MS (DX500 – LCQ Fleet with an inverse phase C18 column). On some of the filters, more than twenty-five organic components were detected (glycolic acid, glyoxylic acid, tartaric acid, malic acid, lactic acid, malonic acid, succinic acid, hydroxybutyric acid, methylmalonic acid, fumaric acid, ketobutyric acid, maleic acid, glutaric acid, oxoheptanedioic acid, citraconic acid, methlysuccinic acid, methylglutaric acid, adipic acid, pimelic acid, phtalic acid, pinic acid, isophtalic acid, suberic acid, benzoic acid, azelaic acid, and sebacic acid). Total mercury measurements from filter samples were performed with a DMA-80 (Milestone) analytical system based on the principles of sample thermal decomposition, mercury amalgamation and atomic absorption detection. Additionally, filters collected at AMS, CPT, PDD, and PDM sampling sites were analyzed by LC-MS technique. Except for total mercury measurements (which were performed at GET, Toulouse), all analyses were performed at the AirOSol chemical analytical platform facility at IGE, Grenoble, France.

## Results

For most of the sites, the TB were in the range of 10^1^–10^2^ 16S rRNA copies per mm^2^ (Table 2). A few high outliers remained and they could be attributed to the cleaning and packing procedures, and to the DNA extraction (including possible cross contamination during the subsampling phase). FB consisting of filters exposed to the atmosphere for up to 1 week but with no air forced to pass through, had, as expected, 16S rRNA gene concentrations one-to five-fold higher than the TB. These combined the passive contribution of the atmospheric environment and the DNA contamination occurring during the different phases of filter handling in the field. Except for the polar sites and CHC, the concentration of 16S rRNA gene copies in blank samples were < 0.3% that in the corresponding atmospheric samples. The blanks at CHC were up to 7% of the average number of copies in the atmospheric samples, due to the low concentrations of DNA sampled from air at this high altitude site. At both polar sites (DMC and Villum) the 16S rRNA gene concentrations were similar to controls, indicating very low biomass.

**Table 2:** Number of 16S rRNA gene copies per mm^2^.

Site code	Sampling site location	Type of sample	*n*	Minimum value	Maximum value	Mean	*SD*
AMS	Amsterdam Island, France	TB	2	29	41	35	8
		FB 72 h	2	85	115	100	21
		Sample	9	2941	82353	45686	26521
CPT	Cape Point Station, South Africa	TB	2	29	65	47	25
		FB 72 h	2	41	47	44	4
		Sample	7	4118	94118	42689	39281
CHC	Chacaltaya, Bolivia	TB	2	79	274	176	137
		FB 72 h; FB 168 h	3	588	1088	838	354
		Sample	16	353	32353	11928	10088
DMC	Concordia Station, Antarctica	FB	2	41	76	59	25
		Sample	3	29	71	53	21
GRE	Grenoble, France	TB	NA	NA	NA	NA	NA
		FB 72 h	2	31	126	79	68
		Sample	10	12647	705882	326242	270626
NAM	Nam Co, China	TB	2	941	1471	1206	374
		FB 24 h	1	–	–	233	–
		Sample	9	21765	882353	355686	304699
PDD	Puy de Dôme, France	TB	8	21	1765	477	639
		FB 48 h; FB 144 h	2	143	882	401	417
		Sample	63	94	58823529	1418588	7510189
PDM	Pic du Midi, France	TB	3	40	94	58	31
		FB 168h	1	–	–	107	–
		Samples	14	1176	64706	21443	18917
STN	Villum research Station, Station Nord, Greenland	TB	2	74	235	154	114
		FB 72 h	2	59	529	294	333
		Samples	13	29	882	222	299
STP	Storm Peak Laboratory, United States	TB	2	44	88	66	31
		FB 72 h	2	176	471	324	208
		Samples	7	706	441176	230353	177472

*TB, transportation blanks; FB 72h, field blanks exposed for 72 h. NA indicate non-available measurements*.

Organic carbon results (Table 3) also confirmed that the sampling protocols were adequately designed with a mean OC value of 0.55 ± 0.26 μg/cm^2^ for all the transportation blanks, and of 0.85 ± 0.32 μg/cm^2^ for the field blanks. In the case of the PDD samples, TB or FB represented less than 1.5% of the OC content in a sample. At remote sites with a very low OC concentration such as at AMS, the FB fraction reached up to 30% of the value in samples, but was thus still clearly distinct. Based on our experience in atmospheric chemistry field programs (Sprovieri et al., 2016; Daellenbach et al., 2017; Pandolfi et al., 2018), these are low concentrations for blank series. As shown in Figure 3, our protocols significantly lower the transportation blank regarding OC. Inevitably, the handling of the filter clearly induces some contamination. This contamination can be significantly reduced when using a laminar flow hood, as in Grenoble (field blank of 0.49 μg/cm^2^) and PDD.

**Table 3:** Organic carbon concentrations expressed in μg per cm^2^.

	Type of		Minimum	Maximum
Site	sample	*n*	value	value	Mean	*SD*
AMS	TB	2	0.45	0.51	0.48	0.04
	FB 72 h	2	0.54	1.10	0.82	0.39
	Sample	9	2.13	3.89	2.83	0.63
CPT	TB	2	0.69	0.72	0.71	0.01
	FB 72 h	2	0.87	1.29	1.08	0.29
	Sample	7	5.77	10.69	7.93	2.20
CHC	TB	2	0.21	0.23	0.22	0.01
	FB 72 h; FB 168 h	2	0.49	0.59	0.54	0.05
	Sample	16	4.53	12.09	8.10	2.34
DC	FB	2	NA	NA	NA	NA
	Sample	3	NA	NA	NA	NA
GRE	TB	2	0.27	0.28	0.28	0.01
	FB 72 h	2	0.37	0.61	0.49	0.17
	Sample	10	73.50	145.65	105.10	21.87
NAM	TB	0	NA	NA		NA
	FB 24 h	1	NA	NA	0.99	NA
	Sample	9	3.97	12.09	8.50	2.87
PDD	TB	6	0.45	1.02	0.67	0.21
	FB 48 h; FB 144 h	5	0.53	1.28	0.90	0.25
	Sample	63	13.63	166.50	68.53	35.91
PDM	TB	2	0.74	0.84	0.80	0.05
	FB 168h	1	–	–	1.22	–
	Samples	14	4.47	32.47	19.02	7.02
STN	TB	2	0.64	1.04	0.83	0.10
	FB 72 h	2	1.20	1.34	1.27	0.28
	Samples	13	1.87	9.66	5.01	2.41
STP	TB	2	0.29	0.32	0.31	0.01
	FB 72 h	2	0.25	0.75	0.50	0.25
	Samples	7	3.18	108.75	70.10	36.82

*TB, transportation blanks; FB 72 h, field blanks exposed for 72 h. NA refers to non-available data due to the absence of analytical data for this particular filter*.

**Figure 4:**
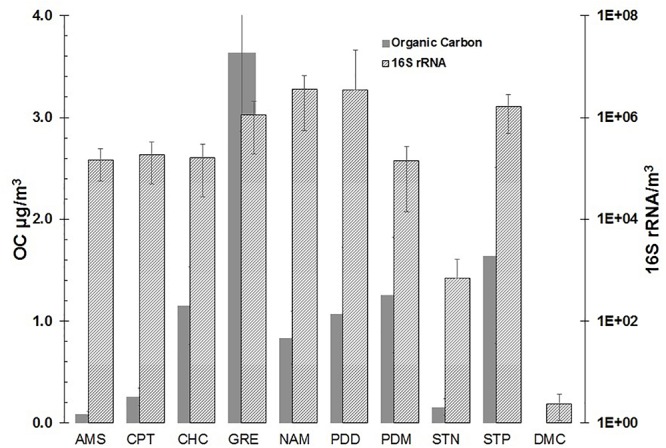
OC and 16S rRNA gene concentrations (mean ± SD) measured at the different sampling sites.

Figure 4 reports the range of OC and 16S rRNA gene concentrations measured at the different sampling sites. As expected, the urban site (Grenoble) had the highest OC values (3.63 ± 0.78 μg/m^3^) and 16S rRNA gene concentrations, of around 10^6^ copies/m^3^ of air (1.1 ± 0.9 × 10^6^). Comparable gene copies concentrations were observed at Storm Peak (1.6 ± 1.1) 10^6^ copies/m^3^, Nam Co (3.6 ± 3.1) 10^6^ copies/m^3^, but lower OC values comparatively to the Grenoble site.

This indicates that DNA material has, at least in part, sources distinct from OC. Puy de Dome station, where 53 samples were collected over 1 year, showed a range of gene copies from 10^4^ to 10^8^ copies/m^3^ ((3.4 ± 18) 10^6^ copies/m^3^ in average (±standard deviation) illustrating the great temporal variability of airborne biological material in the air at a single site. This is to be related with the wide diversity of air masses and meteorological conditions that can occur at a given site over a year (see for example Deguillaume et al., 2014). More remote sites had all 16S gene concentrations one order of magnitude lower than at PDD, such as Chacaltaya (1.6 ± 1.3) 10^5^ copies/m^3^ (night samples only), Cape Point (1.9 ± 1.4) 10^5^ copies/m^3^, Pic du Midi (1.4 ± 1.3) 10^5^ copies/m^3^ and Amsterdam Island (1.5 ± 1.0) 10^5^ copies/m^3^. In turn, the OC concentrations were clearly distinct between the sites, with Amsterdam Island showing the lowest OC levels of this study (0.09 ± 0.02) μg/m^3^. The arctic site had a very low 16S rRNA gene concentration of 10^2^/m^3^ for corresponding average OC concentrations of 1.6 ± 0.9 μg/m^3^, while the Antarctic site on the plateau showed gene copies similar to the blank.

## Conclusion

We developed suitable easy-to-use and standardized protocols that generated consistent microbial and chemical datasets from the same samples at concentrations high enough for confident analysis. The validity of these protocols was then tested by deploying them on 10 sites over the globe and collect samples to explore atmospheric bacteria, fungi, or viruses over large spatial scales. For large scale studies coupling chemical measurements to biological measurements, we were able to demonstrate that quartz filters can be used, but that the extraction protocols must be optimized to maximize DNA yield. In addition to using the protocol outlined here, we also recommend the following:

• carefully prepare the filter and all the material using a combination of heating (500°C) and UV treatment (254 nm).
• provide detailed protocols intended to limit contamination (SOP, see SI for an example) to field users.
• include enough control samples (including transportation and field work blanks) to monitor the quality of the sampling procedure.
• carefully design atmospheric sampling at peak stations in order to take into account vertical turbulent mixing, and night-time hours (in general) should be preferred to avoid the influence of local sources of aerosols.
• Correct sampling times for the remoteness of the sites and for the measurements to be carried out. For example, a 1 week sampling with a volume of around 5000 (normalized) m^3^ is sufficient for amplicon and metagenomic sequencing for most remote sites, except Antarctica, where the biomass is too low for DNA investigations even from total filtered volumes of 16000 m^3^.
• Collected volumes should be normalized using STP or SATP standards.

One of the main disadvantages of a weekly sampling is the loss of information regarding rapid atmospheric chemistry processes and rapid changes in terms of aerosol sources. In turn, it has the advantage to smooth the data and avoid the stochastic-like behavior of biological content in the air often observed (Bowers et al., 2009, etc.). With the development of better extraction protocols and more sensitive sequencing techniques, this limitation could be overcome, allowing for daily sampling in the future.

## Author Contributions

AD, CathL, TV, and PA conceived the study. OM implemented the technical phase and protocols together with AT and RT-P. RT-P performed the biomolecular work. J-LJ supervised the analytical work. MJ, LB, KS, JS, MA, IM, CasL, LM, and QZ helped and participated to the implementation of the field experiments.

## Conflict of Interest Statement

The authors declare that the research was conducted in the absence of any commercial or financial relationships that could be construed as a potential conflict of interest.
