# Supplementary material for: Methods to Investigate the Global Atmospheric Microbiome
Source: Front Microbiol. 2019 Feb 21;10:243. doi: 10.3389/fmicb.2019.00243 (PMC6394204; doi:10.3389/fmicb.2019.00243)
Supplement: Supplementary file 1 [file Data_Sheet_1.PDF]

Before starting the experiment: PLEASE CONTACT US
xxxxx : xxxxx@xxxx

POTOCOL – FILTER PREPARATION

RECOMMANDATIONS:
- Never touch the filter with your bare hands.
- Don't cough or sneeze on the filter.
- Work as cleanly as possible.

MATERIAL THAT YOU NEED:
- A NEW pair of gloves
- 2 NEW plastic tweezers
- A NEW Filter
- A pre-labeled sterilized plastic bag for the transport of the new filter in the older filter

AT THE LAB, under a laminar flowhood or on a clean bench if any.

1. PUT A NEW PAIR OF GLOVES.
2. Sterilize each parts of the older filter 2min under the Black UV light.

   Be careful with the UV lamp !!
3. Take a new pair of tweezers (or sterilize the metal tweezers) and put a new filter on the older filter.

   Be careful, you need to expose to the air the side with relief of the filter.
4. Close the older filter and put it in a new plastic bag used to the transport to the sampler.
5. Change the filter of the impactor with a new one.
6. You can keep the same gloves and go to the sampler to begin a new sample.

PROTOCOL – FILTER STORAGE

RECOMMANDATIONS:

- Never touch the filter with your bare hands.
- Don't cough or sneeze on the filter.
- Work as cleanly as possible.

MATERIAL THAT YOU NEED:

- A NEW pair of gloves
- 2 NEW plastic tweezers
- A sterilized aluminum foil
- A pre-labeled sterilized plastic bag corresponding to the sampler just collected

AT THE LAB, under a laminar flowhood or on a clean bench if any.

1. PUT A NEW PAIR OF GLOVES.
2. Sterilize the 2 pliers
3. Remove the rings of the filter holder using the tweezers and dispose it on the clean working surface. Please, DON'T touch the filter, even with gloves !
4. Unload the Quartz filter (= the sample) using the two pliers on the non-impacted parts and put it on the aluminum foil, fold the exposed portion upon itself (picture 1a). Don't touch it with your gloves, just with the pliers.
5. Fold to close the aluminum foil (making several folds, see picture 1b).

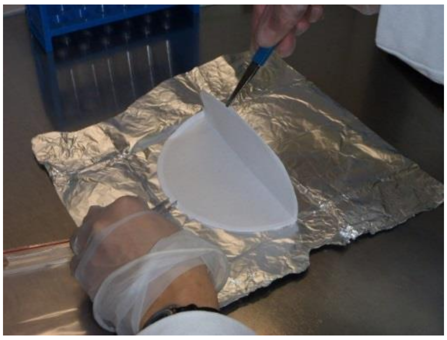

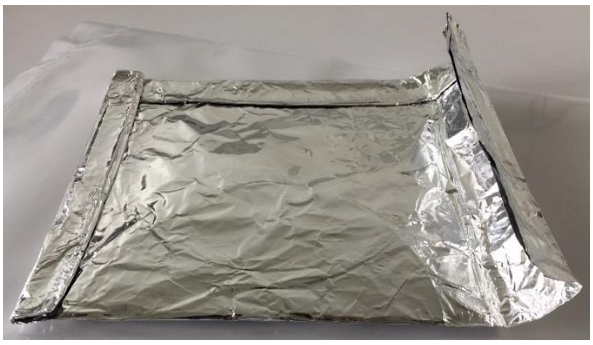

Pictures 1a (left) and 1b (right): Folding of the filter & Folds of the aluminum foil

6. Put it in the plastic bag dedicated to this sample.
7. Note the characteristics (Date, Operator, any useful remarks) of this sample on the pre-identified paper wich is inside the plastic bag.

   Close the plastic bag using a heat sealer and put it in the freezer (<-10°C or below). If you don't have a heat sealer, use a stapler.

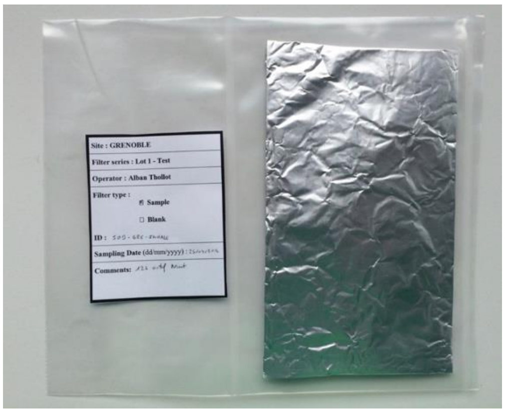

Picture 3: Plastic bag with the sampler

8. On the computer, write the characteristics of the samples (old and new one) in the Excel file and send EACH WEEK after the collect this file with the data (ex: S01.CSV, S02.CSV,…) by email at: xxx@xxx

THANK YOU VERY MUCH !!
